# MASSIF-1: a beamline dedicated to the fully automatic characterization and data collection from crystals of biological macromolecules

**DOI:** 10.1107/S1600577515016604

**Published:** 2015-10-03

**Authors:** Matthew W. Bowler, Didier Nurizzo, Ray Barrett, Antonia Beteva, Marjolaine Bodin, Hugo Caserotto, Solange Delagenière, Fabian Dobias, David Flot, Thierry Giraud, Nicolas Guichard, Mattias Guijarro, Mario Lentini, Gordon A. Leonard, Sean McSweeney, Marcus Oskarsson, Werner Schmidt, Anatoli Snigirev, David von Stetten, John Surr, Olof Svensson, Pascal Theveneau, Christoph Mueller-Dieckmann

**Affiliations:** a European Molecular Biology Laboratory, Grenoble Outstation, 71 avenue des Martyrs, F-38042 Grenoble, France; b Unit for Virus Host Cell Interactions, Université Grenoble Alpes-EMBL-CNRS, 71 avenue des Martyrs, F-38042 Grenoble, France; c European Synchrotron Radiation Facility, 71 avenue des Martyrs, F-38043 Grenoble, France

**Keywords:** automation, beamline, automatic data collection, radiation damage

## Abstract

MASSIF-1 (ID30A-1) is an ESRF undulator beamline operating at a fixed wavelength of 0.969 Å (12.8 keV) that is dedicated to the completely automatic characterization of and data collection from crystals of biological macromolecules. The first of the ESRF Upgrade MASSIF beamlines to be commissioned, it has been open since September 2014, providing a unique automated data collection service to academic and industrial users. Here, the beamline characteristics and details of the new service are outlined.

## Abstract 2

MASSIF-1 (ID30A-1) is a new beamline dedicated to the completely automatic characterization and data collection from crystals of biological macromolecules.

## 1. Introduction   

MASSIF-1 is a new beamline dedicated to the fully automatic characterization of and data collection from crystals of biological macromolecules. The first beamline of the ESRF Upgrade UPBL10/MASSIF project (Theveneau *et al.*, 2013 ▸) to be commissioned, it replaces ID14-1 and is based on a similar, but greatly improved, optical setup. The highly successful ID14 complex of beamlines allowed the construction of four beamlines on a single insertion device (Wakatsuki *et al.*, 1998 ▸). The ‘Quadrigia’ concept used synthetic diamonds (asymmetric Laue [100]) to produce three fixed-wavelength beamlines (ID14-1, ID14-2 and ID14-3) and a tunable high-flux MAD beamline (ID14-4; McCarthy *et al.*, 2009 ▸). While highly productive, the configuration had disadvantages in that absorption from the diamonds reduced the flux available on ID14-4 at longer wavelengths and changing energy on ID14-4 affected the flux available on the other beamlines. ID14 acted as a test bed for the automation of macromolecular crystallography (MX) experiments, seeing the first robotic sample changers to be installed in Europe (Cipriani *et al.*, 2006 ▸) and, along with the other ESRF MX beamlines, pioneered the pre-data collection screening and evaluation of samples (Bowler *et al.*, 2010 ▸; Brockhauser *et al.*, 2012 ▸; Incardona *et al.*, 2009 ▸). The level of automation on the ESRF MX beamlines led to their heavy use for the most challenging projects in structural biology, and in many cases the evaluation of the diffraction properties of large numbers of crystals on ID14-1, ID14-2 and ID14-3 was performed before full data collection on the best samples of a particular project was carried out on ID14-4 or other high-flux [ID23-1 (Nurizzo *et al.*, 2006 ▸), ID29 (de Sanctis *et al.*, 2012 ▸)] or microfocus (ID23-2; Flot *et al.*, 2010 ▸) ESRF MX endstations. This way of using the ESRF’s MX facilities stimulated the design of a new suite of beamlines that would have the density of ID14 but with greater independence coupled to second-generation automation. The ESRF Upgrade Program Beamline UPBL10 was proposed to address the problem of crystal evaluation with the Massively Automated Sample Selection Integrated Facility (MASSIF), the replacement for ID14 located at ID30. The core of the facility is composed of three beamlines, two (MASSIF-1 and MASSIF-2) using diamond beam splitters, as on ID14, and a third microfocus beamline (MASSIF-3) using a Si[111] single-bounce monochromator, with the central philosophy of maximum automation (Theveneau *et al.*, 2013 ▸). In addition, a fully tunable MAD beamline to replace ID14-4, ID30B, is currently being commissioned. As ID30 has canted undulators, the MASSIF branch is independent of the tunable branch which thereby removes the disadvantages of the previous design. MASSIF-1 is the first of these beamlines to be commissioned and has been dedicated to the fully automatic evaluation and data collection from crystals of biological macromolecules. The new service is not designed to replace user visits to the synchrotron, but rather to do the hard work of screening crystals or collecting data sets through the night, freeing researchers to spend time on more challenging data collection problems and study the underlying biology.

## 2. Beamline overview   

**Figure 1:**
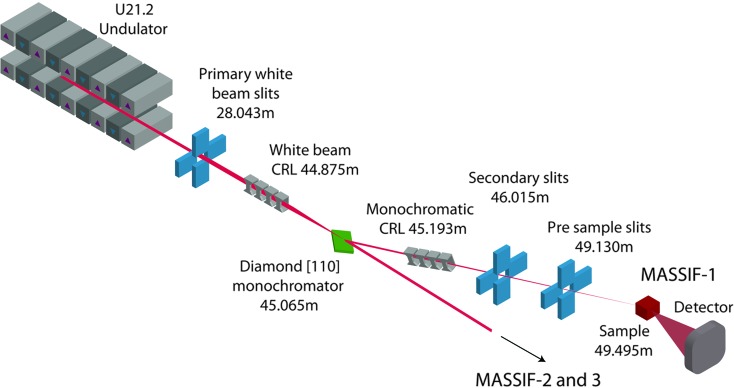
Schematic of the MASSIF-1 layout. Major components are shown with their distances from the source.

The MASSIF complex is situated on a new ESRF 6 m straight section at ID30 which allows the provision of two independent branches using a canted approach, without compromising undulator length. The MASSIF branch (ID30A) uses two 1.4 m undulators with a periodicity of 21.2 mm (U21.2) operated in air with a minimum gap of 11 mm and their highest brilliance at an energy of 12.9 keV; thereby allowing the anomalous diffraction from selenium and other heavy atoms to be exploited in diffraction experiments aimed at *de novo* structure solution using a remote SAD approach (Leonard *et al.*, 2005 ▸). As automation is the central requirement of the beamline, a simple optical setup was selected in order to increase the reliability of the beam position and reduce the number of components that require alignment. MASSIF-1 uses a synthetic asymmetric Laue [110] diamond as a monochromator and a compound refractive lens (CRL; Snigirev *et al.*, 1996 ▸; Vaughan *et al.*, 2011 ▸) as the sole focusing element. A white-beam CRL used for the vertical focusing for MASSIF-3 also provides some pre-focusing for MASSIF-1. The endstation layout is summarized in Fig. 1 ▸ and Table 1 ▸. In order to automate and facilitate beam alignment, beam position monitors have been placed before and after most optical elements.

**Figure 2:**
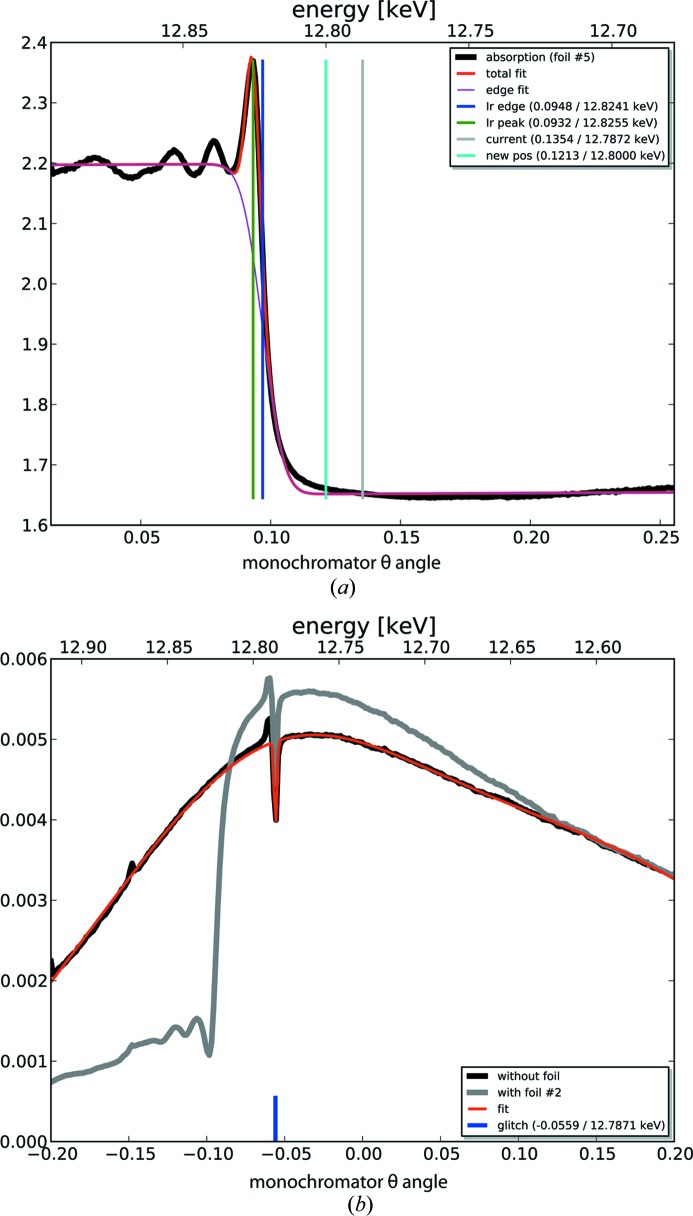
Calibration of the MASSIF-1 Laue [110] diamond monochromator. (*a*) Scan in transmission mode of the Ir *L*_II_ absorption edge. The blue line shows the position of the inflection point, the cyan line the angle to which the monochromator is moved to ensure a monochromatic X-ray beam of *E* = 12.80 keV (λ = 0.969 Å). (*b*) Scan of the MASSIF-2 monochromator θ angle; the glitch from the MASSIF-1 monochromator can be seen at 12.78 keV before it was moved to the calibrated position. Intensity units are arbitrary.

The use of artificial thin diamond crystals has been essential in maximizing the exploitation of photons produced at ID30A. However, while asymmetric Laue [100] diamonds served ID14 well, advances in the production of diamonds mean that new options are available which increase the available flux at the sample position for multiple endstations based on beam splitters. In this regard, the most desirable diamonds are Bragg [111], but as these are not yet available a Laue [110] diamond is used for MASSIF-1. The advantage of the Laue [110] geometry is that, in order to place the Bragg planes to diffract the white beam, the angle required is near 90° to the plane of the crystal, almost optimal for maximum transmission of the white beam to the downstream endstations MASSIF-2 and MASSIF-3. The diamond is mounted in a water-cooled copper support with vertical and horizontal translation, rotation of θ with a precision of 1 eV (4 µrad) as well as the ability to tilt the vessel. The energy of the X-ray beam is calibrated by introducing an iridium foil and diode behind the monochromator and scanning (in transmission mode) over the Ir *L*_II_ absorption edge. The resulting scan (Fig. 2 ▸ *a*) gives the position of the absorption edge (*E* = 12.82 keV) as a function of monochromator angle (θ) which is then used to calibrate the working energy of the beamline to 12.8 keV. Scanning the downstream monochromator of MASSIF-2 (diamond [110]) shows a glitch at the operating energy of MASSIF-1, with a width of *ca* 5 eV (Fig. 2 ▸ *b*). This glitch can be used as an internal control for the calibration of the monochromator.

**Figure 3:**
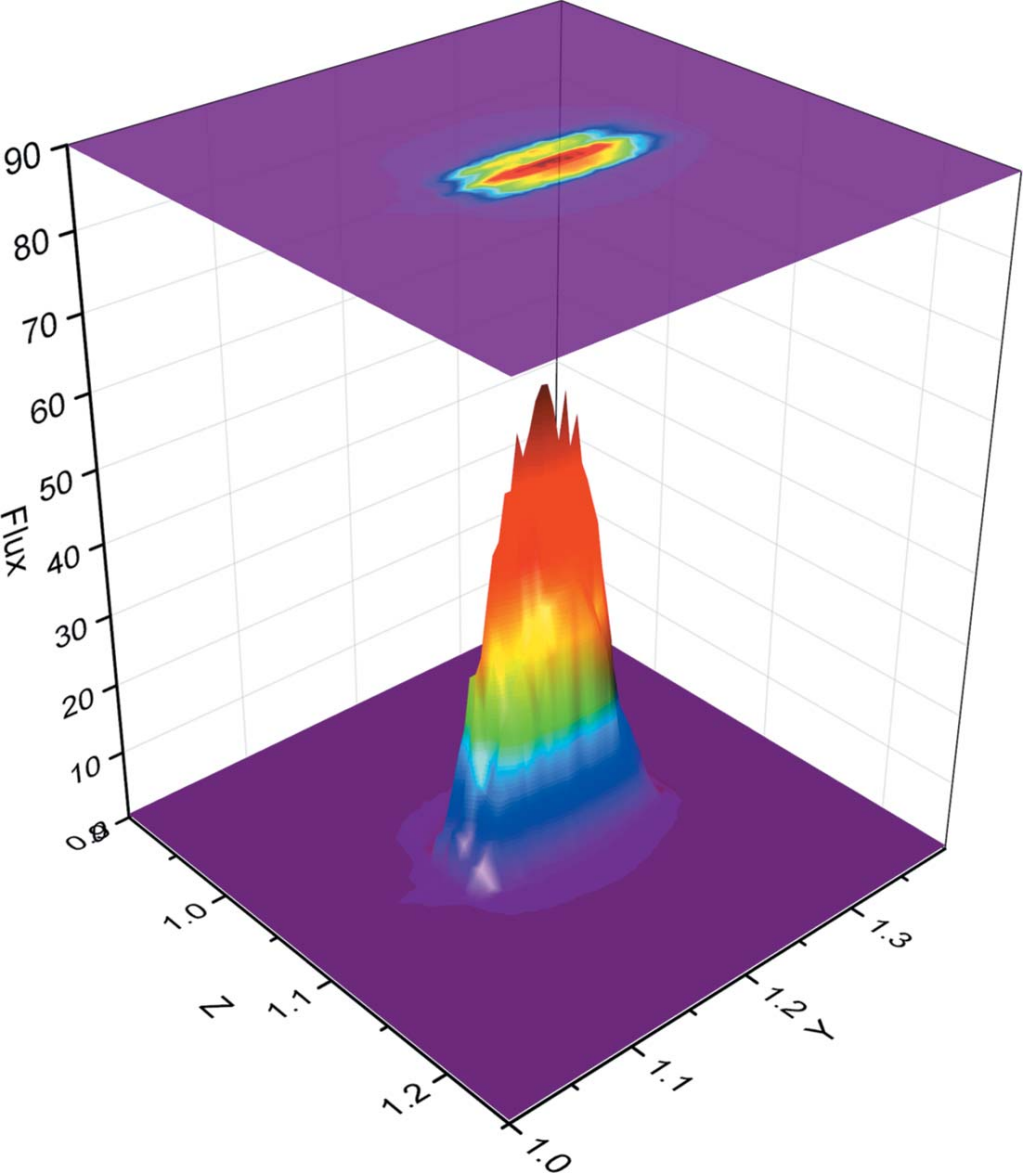
Three-dimensional profile of the beam at the sample position. The two-dimensional profile is shown above. The flux is shown in arbitrary units.

**Figure 4:**
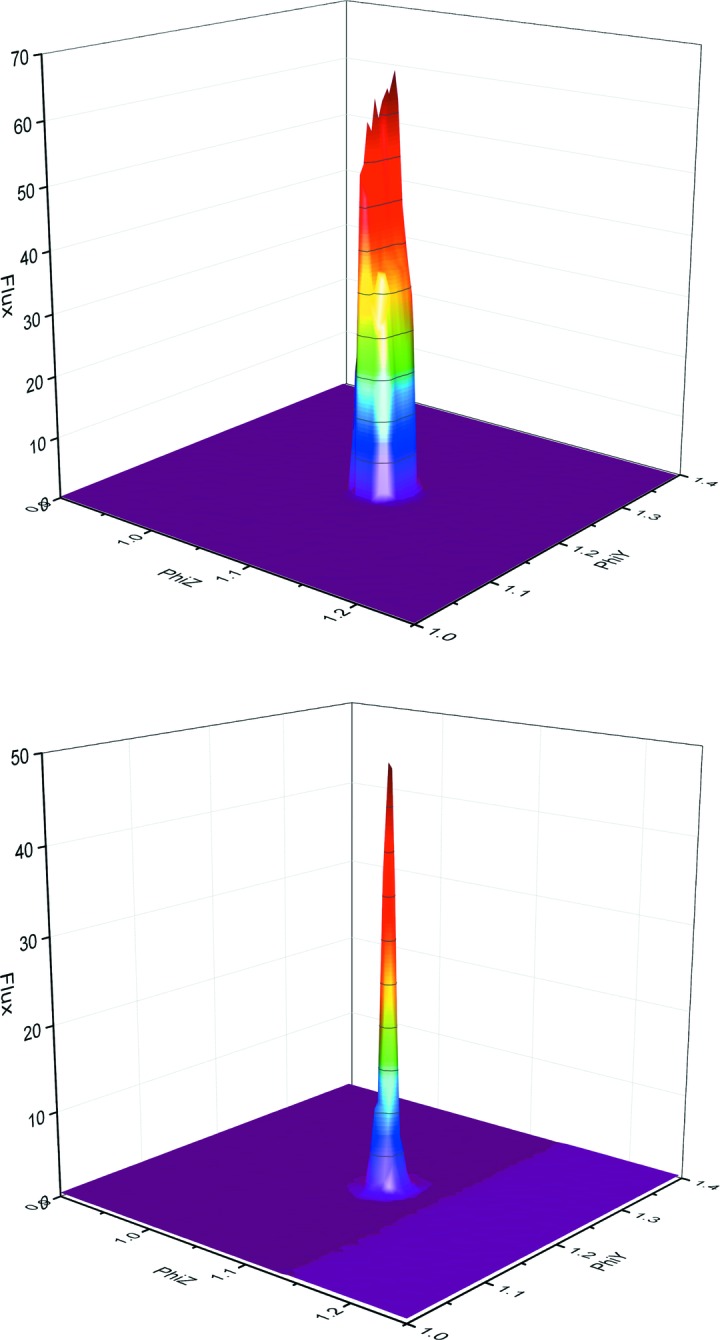
Three-dimensional profile of the beam when using apertures. The 50 µm (top) and 10 µm (below) profiles are shown; 30 µm, 20 µm and 15 µm apertures are also available. Using the central part of the full beam Gaussian leads to a near ‘top-hat’ profile of the beam. The flux is shown in arbitrary units.

A transfocator [a CRL assembly in which individual lenses can be pneumatically inserted or removed from the beam path as required (Vaughan *et al.*, 2011 ▸)] is used as the only focusing element for the beam and enables different combinations of horizontal and vertical lenses to be inserted in order to rapidly change the beam size (see supporting movie). The standard configuration places 13 horizontal and seven vertical lenses in the beam path to produce a focal spot of 221 µm × 65 µm (H × V, FWHM) with a flux of 4.5 × 10^12^ photons s^−1^; this is collimated with slits to produce a beam at the sample position of 100 µm × 65 µm (H × V, FWHM; Fig. 3 ▸) with a flux of 2 × 10^12^ photons s^−1^ at a storage ring current of 200 mA. The beam area can be further tailored by the insertion of apertures between 50 and 10 µm in diameter, allowing the beam size to be matched to that of the crystal (Fig. 4 ▸). As with all ESRF MX beamlines the experimental hutch equipment is placed on a granite table facilitating alignment of all elements simultaneously relative to the X-ray beam by scanning horizontal and vertical translations of the table. The X-ray beam is constantly monitored by two scattering foil diodes placed before and after the final slits. The diode reading is regularly correlated to photon flux using a calibrated diode placed at the sample position, providing real time flux measurements. Exposure of the sample to X-rays is controlled by an ESRF-developed stepper-motor fast shutter that is synchronized to the goniometer axis.

## 3. Ancillary facilities   

As users are not present at the beamline, laboratory facilities are not required. However, provision has been made to store Dewars near the beamline and systems are in place to help users with the tracking of Dewars to and from the beamline, the easy upload of information required for data collection and fast access to results from the beamline *via* the laboratory information management system (LIMS) ISPyB (Delagenière *et al.*, 2011 ▸).

### 3.1. Beamline control   

MASSIF-1 is the first beamline at the ESRF to use a new sequencer based on Python in place of SPEC. This open-source software project, code-named ‘Khoros’ (https://github.com/mguijarr/khoros), uses the latest libraries developed within the ESRF Beamline Control Unit and is the forerunner of a new control software package that will eventually be deployed on all ESRF beamlines. The main parts of the new system are: Beacon (https://github.com/mguijarr/beacon), a beamline configuration library; Emotion (https://github.com/esrf-emotion/emotion), the ESRF library for motor controllers; a set of beamline components, representing real devices that can be directly manipulated from Python code; core functions to perform scanning and data acquisition and a command-line interface that is either terminal-based or *via* a web application shell.

The Khoros project is organized as a Python package, with well defined modules, allowing it to be used as a Python extension library and be embedded into any Python application. On MASSIF-1, Khoros is embedded within the MXCuBE graphical application (Gabadinho *et al.*, 2010 ▸). Data collection routines previously written as SPEC macros are now written as Python functions. As Python is a widely used language for scientific applications, this provides access to thousands of Python modules, powerful data types (*e.g.* list, dict, set) and easy sharing of sequences between facilities, as is the case with the MXCuBE collaboration.

Khoros relies on the latest Python extension modules from industry, notably Gevent (http://www.gevent.org) for asynchronous network communication and co-routine support. The entire project is based around the idea of event-driven asynchronous programming, balanced with the use of co-operative code execution in co-routines. This means that code is simplified and more reliable, as problems of multi-threaded programming are eliminated and both reading and writing of sequences by users is facilitated. As a consequence, Khoros has proved to be a stable and efficient platform on which to build the MASSIF-1 control system.<!?tpb=-12pt>

### 3.2. The RoboDiff and high-capacity Dewar   

**Figure 5:**
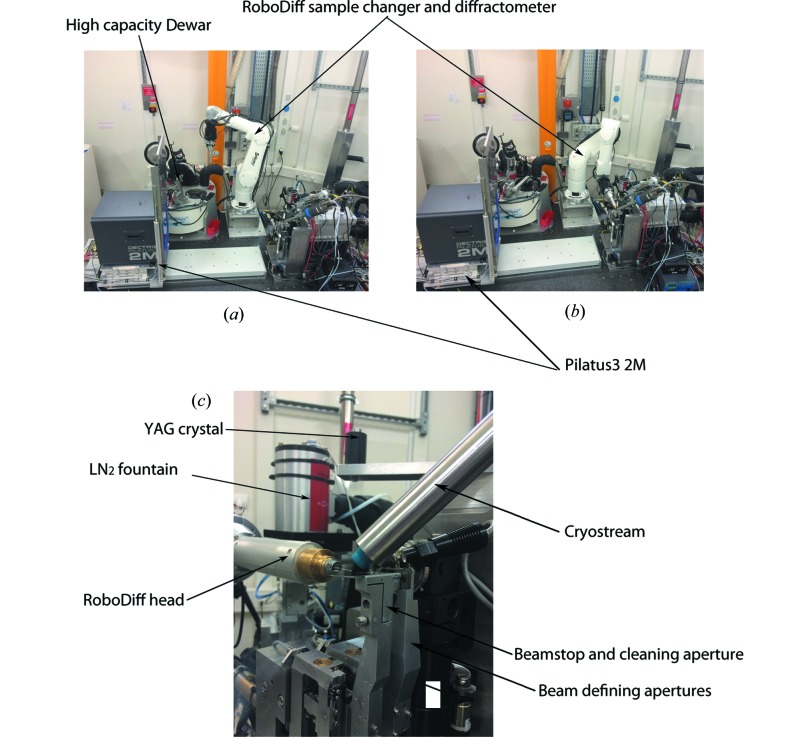
Experimental hutch configuration. The main components of the MASSIF-1 experimental hutch are shown with the RoboDiff in parked (*a*) and goniometer positions (*b*). (*c*) A close-up of the MASSIF-1 sample environment with the RoboDiff in goniometer position.

The beamline is equipped with a robotic sample changer that also acts as a high-precision goniometer, named RoboDiff, developed at the ESRF; it will be described in detail elsewhere (Nurizzo *et al.*, 2015 ▸). The system is based on an industrial six-axis robotic arm. The use of industrial robotics brings reliability and accuracy (repeatability of ∼20 µm) but lacks the high level of precision required for mesh scans and rotations that are needed for small protein crystals (Ferrer *et al.*, 2013 ▸). Therefore, the robot axes are used solely for the purpose of transferring samples between the Dewar to the sample position, and a high-precision *xy* table combined with an air bearing with a sphere of confusion below 2 µm are used for sample manipulations in the X-ray beam (Fig. 5 ▸). Crystals are transported to the sample position in SPINE standard sample holders, the vial of which is removed by an assistant robot arm. Error handling, including automatic recovery and trouble shooting, is essential in order to reduce maintenance and technical interventions, which can reduce the throughput of the beamline. Samples are tracked from the Dewar to the beam position and maintained at liquid-nitrogen temperature to avoid crystal damage by a rapid change of temperature. A liquid nitrogen fountain allows the filling of vials for the preservation of crystals when unmounting (Fig. 5 ▸ *c*). Sensors are used to detect a sample on the robot head and a vial in the assistant so that many problems, including the loss of a sample during a transfer or the loading of a sample when one is already in place, can be avoided. Object recognition cameras are used at major points of the trajectories, such as the exit of the Dewar or at the beam position, for vial recognition. The information provided allows trouble-shooting and automatic recovery procedures to run. The most commonly encountered errors arise when the assistant cannot remove a vial or a vial is poorly placed on the cap for replacement in the Dewar. Error handling means that for the first case the sample is returned safely to the Dewar and the system can move to the next sample. In the latter case, the cap and vial are placed in a bin to prevent a crash in the Dewar. Occasionally, a cap is removed without a vial; in these cases the cap is placed in the bin immediately and the next sample loaded. These errors account for less than 2% of the samples processed so far and, as only one case results in a lost sample without data collection, the error rate can be regarded as lower.

In order for the beamline to work autonomously for long periods a sample storage Dewar is required that will take a large number of samples without manual feeding being required. To this end, the high-capacity Dewar (HCD; Fig. 5 ▸) was designed to take 24 EMBL/ESRF pucks each taking ten SPINE standard samples (Cipriani *et al.*, 2006 ▸) allowing the beamline to run for up to 24 h without manual intervention. The essential design features are high-precision movement, the placement of most mechanical parts out of the liquid nitrogen and the avoidance of ice build-up from an overpressure of dry nitrogen gas in the Dewar.

### 3.3. Workflows for automatic data collection   

**Figure 6:**
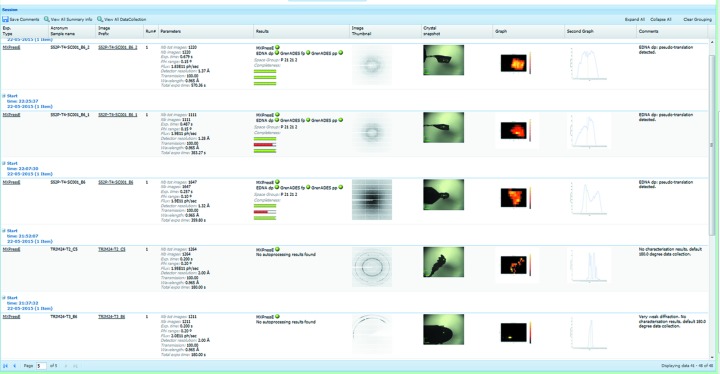
ISPyB summary page. A screenshot showing the display in ISPyB of results, such as diffraction maps, line scans for centring, centring snap shots, diffraction images and the results of autoprocessing, for a series of samples processed using the fully automatic protocols available at MASSIF-1. Comments are automatically written (far right column) to inform users on various stages of the process such as ‘weak diffraction’, default 180° data collection’ *etc*.

As with the other MX beamlines at the ESRF (Flot *et al.*, 2010 ▸; McCarthy *et al.*, 2009 ▸; Nurizzo *et al.*, 2006 ▸; de Sanctis *et al.*, 2012 ▸; Mueller-Dieckmann *et al.*, 2015 ▸), interaction with the beamline components is controlled *via* the GUI MXCuBE2 (Gabadinho *et al.*, 2010 ▸). In combination with the LIMS ISPyB (Delagenière *et al.*, 2011 ▸) and workflows for the automation of data analysis (Brockhauser *et al.*, 2012 ▸; Bourenkov & Popov, 2010 ▸; Incardona *et al.*, 2009 ▸), a fully automatic system has been developed to mount, locate, centre to the optimal diffraction volume, characterize and collect, if possible, diffraction data from a series of cryocooled crystals contained in the HCD (Svensson *et al.*, 2015 ▸). Using a fast X-ray-based routine, samples are located and centred systematically at the position of highest diffraction signal and important parameters for sample characterization, such as flux, beam size and crystal volume, are automatically taken into account, ensuring calculation of optimal data collection strategies. The automatic routines developed are often able to locate crystals more effectively than the human eye either as they are mounted in opaque medium or a large excess of liquid causes refraction and reflection effects. Thus, in many cases, diffraction data have been obtained when centring a crystal was not possible manually. Moreover, for crystals larger than the X-ray beam, all positions within a sample can be evaluated for diffraction quality and this can result in higher-quality diffraction data than might otherwise be the case (Bowler & Bowler, 2014 ▸; Bowler *et al.*, 2010 ▸). Using continuous readout scans to locate and centre crystals, the system operates at about the same speed as a human operator performing only centring and data collection, taking an average of six minutes per sample, depending on sample size and the level of characterization required. Most time is spent on the mesh scan and data collection with average times of just over 100 s each (Svensson *et al.*, 2015 ▸). Users upload essential information *via* ISPyB such as the protein being studied (required by the ESRF Safety Group), a unique sample name and other information to tailor the experiment to the sample. The workflows then use this information and users view a summary of all the results of sample processing (mesh scans, processed data *etc*.) in a summary view in ISPyB (Fig. 6 ▸). Once data sets have been collected they are automatically processed using routines (Monaco *et al.*, 2013 ▸) based on XDS (Kabsch, 2010 ▸) allowing users to quickly see the essential characteristics of a particular data set. Data sets are also analysed for the presence of anomalous scattering and, if present, a structure solution pipeline is launched (Monaco *et al.*, 2013 ▸). The X-ray centring routine used for MASSIF-1 has been exported to all ESRF MX beamlines and is currently the most frequently used workflow by users present at the beamlines.

## 4. Facility access   

**Figure 7:**
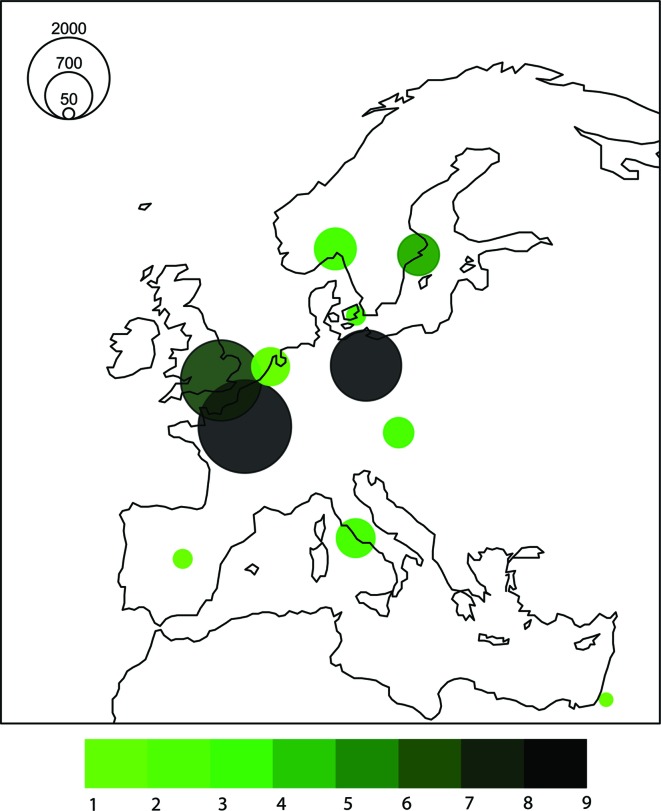
Distribution of crystals sent to MASSIF-1 from across Europe in the first eight months of operation (September 2014–June 2015). Circles are scaled to the number of crystals and are centred on the capital city of the country from which the crystals were sent. The colour is scaled to the number of groups using the beamline.

MASSIF-1 is a fully automated beamline and users are never present at the beamline. A flexible booking system has been introduced where slots can be booked based on the number of samples in an online calendar, rather than the rigid scheduling in place for other MX beamlines. When the samples arrive they enter a queuing system and results are guaranteed within three working beam days of the allotted slot. The number of samples processed on the beamline is then converted into shifts and counted against allocated beam time for a Block Allocation Group (BAG). Applications can also be made *via* a rolling process (see http://www.esrf.eu/MXnon-BAGproposal) and once shifts have been allocated slots are booked on-line in the same manner as for BAGs. The beamline is also heavily used for proprietary research by the pharmaceutical and biotechnology industries. The flexibility in scheduling is highly appreciated by the user community. Since opening in September 2014, MASSIF-1 has processed over 10000 crystals from across Europe (Fig. 7 ▸) without any human intervention.

## 5. Highlights   

**Figure 8:**
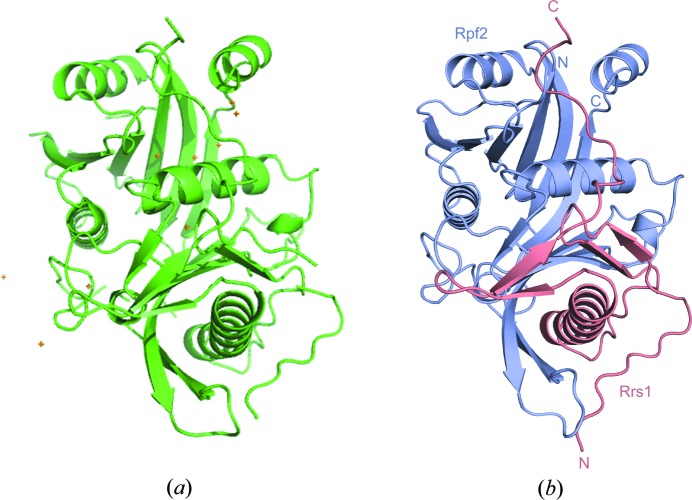
The Rpf2-Rrs1 complex structure solved using diffraction data collected automatically on MASSIF-1. (*a*) The model determined by the automatic SAD pipeline is shown as a cartoon from the Cα trace with the Se atom substructure shown as gold crosses. (*b*) The final refined model is shown with Rpf2 in magenta and Rrs1 in salmon.

The beamline has been open since September 2014 and to date has processed over 10000 samples. This throughput has now started to result in publications based on diffraction data collected on the endstation (Koromyslova *et al.*, 2015 ▸). A recent example highlights the advantages of flexible booking and automated data collection (Kharde *et al.*, 2015 ▸). The Rpf2-Rrs1 complex is thought to have a role in ribosome biogenesis but no structural information has been available for the complex. Initially, 70 crystals of the native complex were obtained and rapidly screened on MASSIF-1. This resulted in ten data sets from crystal forms with different space groups and resolution limits (Table 2 ▸). As molecular replacement failed to produce a successful structure solution, a second session was booked with 40 crystals of the selenomethionine derivative screened. This resulted in ten data sets mostly in a centred orthorhombic space group with the best diffraction to 1.6 Å (Table 2 ▸). These data sets were processed automatically and this resulted in nine of the data sets being phased by SAD protocols (Monaco *et al.*, 2013 ▸). The automatically determined model and the final refined model are shown in Fig. 8 ▸. The best data set has excellent processing, phasing and refinement statistics (Table 3 ▸; Kharde *et al.*, 2015 ▸). The automatic service on MASSIF-1 allowed the researchers to screen a large number of crystals when they became available, through flexible booking of beam time, and rapidly feed results back into the project to improve crystal quality and phase the structure by single-wavelength anomalous diffraction within five weeks.

## 6. Discussion and conclusions   

MASSIF-1 is a unique beamline for the fully automatic characterization and data collection from macromolecular crystals. The service offered provides a new tool for structural biologists to screen initial crystallization hits or collect large numbers of data sets without having to control the endstation themselves. The automatic routines available allow data collection to be performed consistently, taking crystal size and flux into account when calculating data collection strategies. Flexibility is introduced into the system by allowing sample specific parameters to be specified in the database ISPyB. Additional features will soon be added to the workflows, such as allowing the collection from multiple crystals on the same sample support or multiple positions within the same crystal and high-exposure mesh scans in order to screen for weakly diffracting samples. While measurements are currently limited to variations around classic experiments, it is hoped that more complex strategies such as helical data collections (Flot *et al.*, 2010 ▸) and goniometer realignment (Brockhauser *et al.*, 2013 ▸) can be included soon. It is also foreseen to include dehydration experiments in the automated pipeline (Bowler *et al.*, 2015 ▸).

The new level of automation should decrease project lifecycles and in partnership with developments being made in the automatic mounting of crystals (Cipriani *et al.*, 2012 ▸), a fully automatic pipeline from protein to structure can now be envisioned.

## Supplementary Material

Video showing the focusing of the X-ray beam by an increasing number of lenses in the transfocator. DOI: 10.1107/S1600577515016604/ie5144sup1.wmv

## Figures and Tables

**Table 1:** Beamline details

Beamline name	MASSIF-1 (ID30A-1)
Source type	2 × 1.4 m in air U21.2 undulators (minimum gap 11 mm)
Focusing element	Be CRL
Monochromator	C[110]
Energy (keV)	12.8
Wavelength (Å)	0.969
Beam size† (uncollimated, H × V FWHM) (µm)	221 × 65
Beam size† (collimated, H × V FWHM) (µm)	100 × 65
Flux‡ (uncollimated) (photons s^−1^)	4.5 × 10^12^
Flux‡ (collimated, typical) (photons s^−1^)	1.2 × 10^12^
Goniometer	ESRF RoboDiff
Sample mounting	ESRF RoboDiff
Detector type	CMOS Hybrid Pixel
Detector model	Dectris Pilatus3 2M

† Measured by scanning a pinhole of 5 µm diameter vertically and horizontally across the beam.

‡ Measured with a calibrated diode at the sample position at a ring current of 200 mA.

**Table:** All data were collected at λ = 0.969 Å. Note the difference in diffraction quality even between samples adopting the same crystal form. The best data set from each run is shown in bold.

Native	First session	22/01/2015	
TOTAL: 70 crystals were screened with ten data sets automatically processed			
Crystal	Characterization successful	*d*_min_ (Å)	Space group from autoprocessing
RibBio-K1	No	3	*C*2
RibBio-K2	Yes	**2.55**	*P*3_1_12
RibBio-E4	Yes	3.13	*P*4_1_2_1_2
RibBio-E10	Yes	2.79	*P*4_1_2_1_2
RibBio-R10	Yes	2.57	*P*222
RibBio-R9	Yes	3.46	*P*222
RibBio-R8	Yes	3.23	*P*222
RibBio-R7	Yes	4.09	*P*422
RibBio-R4	No	3.19	*P*42_1_2
RibBio-R1	Yes	3.97	*P*4_1_2_1_2

**Table:** 

Se-Met	Second session	03/2015	
TOTAL: 40 crystals were screened with ten data sets automatically processed†			
Crystal	Characterization successful	*d*_min_ (Å)	Space group from autoprocessing
RibBio-S9	No	2.18	*C*222_1_
RibBio-S8	No	2.49	*C*222_1_
RibBio-S7	Yes	2.51	*C*222_1_
RibBio-S6	Yes	2.03	*C*222
RibBio-S4	No	2.44	*C*222
RibBio-S2	No	1.91	*C*222_1_
RibBio-A8	Yes	1.91	*C*222
RibBio-A1	Yes	**1.63**	*C*222
RibBio-T5	Yes	2.94	*P*4_1_2_1_2
RibBio-Y5	Yes	1.76	*C*222

† Nine different initial models were provided by the SAD pipeline.

**Table 3:** Data collection and refinement statistics Statistics for the highest-resolution shell are shown in parentheses. *R*_pim_: precision-weighted merging *R*-factor. CC* is an estimate of the ‘true’ CC1/2 of the data under examination to the unknown true intensities. FOM is figure of merit. DM is density modification. + indicates values at 3.0 Å resolution cut-off.

Resolution range (Å)	42.45–1.52 (1.57–1.52)
Space group	*C*222_1_
Unit cell (Å, °)	49.2, 84.1, 194.3, 90, 90, 90
Unique reflections	61799 (5385)
Multiplicity	4.8 (2.4)
Completeness (%)	98.0 (84.1)
Mean 〈*I*/σ*I*〉	11.6 (0.7)
Wilson *B*-factor (Å^2^)	18.9
*R*_pim_ (%)	4.3 (95.7)
CC*	0.999 (0.675)
Anomalous signal+	2.23
FOM before DM+	0.43
*R*_work_ (%)	14.6 (33.0)
*R*_free_ (%)	19.1 (36.9)
No. of non-hydrogen atoms	3020
Macromolecules	2595
Water	425
*R*_msd_ (bonds, Å)	0.012
*R*_msd_ (angles, °)	1.32
Ramachandran plot (%)	
Favoured	99
Outliers	0.3
Average *B*-factor (Å^2^)	29.1
Macromolecules	27.2
Solvent	41
